# Recent Advances in Bacterial Separation and Enrichment from Blood for the Diagnosis of Bloodstream Infections

**DOI:** 10.3390/s26113371

**Published:** 2026-05-26

**Authors:** Hai-Bo Wang, Zhen-Zheng Zhang, Qing Liu, Hang-Bo Lu, Jian-Hui Jiang, Ru-Qin Yu, Hao Tang

**Affiliations:** State Key Laboratory of Chemo and Biosensing, College of Chemistry and Chemical Engineering, Hunan University, Changsha 410082, China

**Keywords:** bacterial, separation and enrichment, bloodstream infection

## Abstract

In this paper, recent advances (2016–2026) in bacterial separation and enrichment from blood for diagnosis of bloodstream infection (BSIs) through pathogen identification and antimicrobial susceptibility testing (AST) are reviewed. The review centers on sample processing as an indispensable front-end of biosensor and lab-on-chip platforms, since most sensors cannot operate directly in whole blood. Efficient separation and enrichment concentrate extremely low bacterial burdens, remove blood components that interfere with detection, and deliver bacteria in a sensor-compatible format; consequently, diagnostic sensitivity, specificity, turnaround time, and robustness are strongly determined by this step. We first summarize the clinical impact of BSIs and the value of rapid AST for guiding timely, targeted therapy, emphasizing that efficient bacterial isolation from blood is a prerequisite for accurate testing. We then discuss key challenges and recent progress in bacterial separation and enrichment from blood with major approaches, including filtration, centrifugation, functionalized magnetic beads, and microfluidic technologies. These strategies serve as core building blocks that interface with downstream identification and AST methods, supporting integrated biosensors and point-of-care devices. Finally, we outline future directions of bacterial separation and enrichment approaches to improve recovery, purity, integration, standardization, and overall diagnostic performance for BSI workflows.

## 1. Introduction

Bloodstream infections (BSIs) are serious medical conditions that occur when pathogenic microorganisms enter the bloodstream, often leading to systemic inflammation and sepsis [1,2,3,4]. These infections can rapidly progress, resulting in organ dysfunction, septic shock, or even death if not promptly treated. BSIs are linked to significant morbidity and mortality, especially in immunocompromised individuals and patients with pre-existing health conditions. Hence, rapid pathogen identification and antimicrobial susceptibility testing in BSIs are essential for guiding timely and effective treatment decisions. The conventional blood culture method is considered the clinical gold standard, but it requires 48 to 72 h to obtain antibiotic susceptibility results. This delay often leads to empirical use of broad-spectrum antibiotics, increasing the risk of inappropriate treatment and antimicrobial resistance [5]. The lengthy process is due to low bacterial concentrations in early-stage infection (1–100 colony-forming units per milliliter, CFU/mL) and limited blood sample volumes (5–10 mL) [6,7]. Blood samples are first incubated in nutrient-rich culture bottles. Once detectable growth of bacteria occurs (~24 h), an aliquot is plated onto agar for isolation of pure strains (~24 h), followed by pathogen identification and AST. It is worth noting that bacterial burden in bloodstream infections is typically expressed as CFU, but CFU measures only the fraction of bacteria that can grow under routine culture conditions. Blood may also contain dormant cells, particularly viable but non-culturable cells (VBNCs), which remain alive but do not form colonies on standard media, often due to stresses such as antibiotic exposure, nutrient limitation, oxidative stress, or host immune pressure [8,9]. Because current clinical gold-standard methods rely on blood culture and colony recovery, they may miss these non-culturable yet viable cells of VBNCs, leading to underestimation of pathogen burden or delayed diagnosis. This limitation is particularly relevant to emerging separation and enrichment technologies, which can recover intact bacteria regardless of immediate culturability. Accurately confirming VBNC may require a combination of strain specificity (genomic sequencing), viable and culturable cells (flow cytometry and routine culturing), which is expensive and lacks convenience [10]. Future diagnostic workflows should therefore consider integrating bacterial isolation with molecular, imaging-based, or viability-informed detection methods that can capture pathogens beyond conventional colony formation, while also considering validation strategies for VBNC populations.

Building on traditional phenotypic methods such as broth dilution and agar dilution, various rapid antimicrobial susceptibility testing (AST) techniques have been developed, including molecular-based approaches (PCR, sequencing), MALDI-TOF MS, and single-cell methods [7,11,12,13,14,15,16,17,18]. These innovative methods require only a minimal number of bacteria to assess bacterial–antibiotic interactions, allowing for rapid or even culture-free identification and susceptibility testing. As a result, they demonstrate significant clinical potential for rapid diagnosis of BSIs. It is important to point out that a critical prerequisite for such rapid diagnostics is the efficient separation of bacteria from the complex blood matrix. Blood contains large quantities of red and white blood cells, platelets, and proteins, which can interfere with pathogen detection and AST. Therefore, effective separation and enrichment techniques are essential to concentrate bacteria while eliminating these interfering components.

Several review articles in recent years have discussed approaches for bacterial separation from blood by leveraging the distinct physical and chemical properties of blood components [19,20,21]. With advances in biotechnology and microfluidic technologies, several new and practical techniques have emerged to facilitate efficient bacterial separation and improve BSI diagnostics in more recent years. This review aims to summarize and discuss the latest research advances (2016–2026) in the separation and enrichment of bacteria from blood samples, with the goal of enhancing the accuracy and speed of bloodstream infection diagnostics. Given the rapid technological progress in this area, a comprehensive review is urgently needed to help researchers, clinicians, and diagnostic developers understand the current landscape, assess the strengths and limitations of new methods, identify emerging trends and challenges, and guide innovation in rapid BSI diagnosis. A structured literature search was performed in PubMed, Web of Science, and Scopus for articles published from January 2016 to March 2026. Search terms included combinations of bloodstream infection, blood, bacteria, separation, enrichment, sample preparation, filtration, centrifugation, magnetic beads, microfluidics, pathogen identification, and antimicrobial susceptibility testing. We included English-language peer-reviewed studies focused on bacterial separation and/or enrichment from blood or blood-related matrices for downstream identification or AST. We excluded studies on non-bacterial targets, non-blood matrices, papers focused only on downstream detection without relevant sample processing, conference abstracts, editorials, patents, and duplicates. Additional relevant papers were identified by screening the reference lists of key articles and reviews.

## 2. Progress in Blood-Borne Bacterial Separation and Enrichment Using Different Approaches

### 2.1. Bacteria Separation and Enrichment from Blood via Filtration

Filtration is an efficient and straightforward method for separation and enrichment that has been widely applied in many fields. This technique is simple to operate and cost-effective. By taking advantage of the pore size of filter membranes, substances or components of different sizes can be retained, and repeated washing can be performed to thoroughly remove impurities. Table 1 summarizes key physical properties of major blood components compared to bacterial pathogens [22,23,24,25,26].

**Figure 1:**
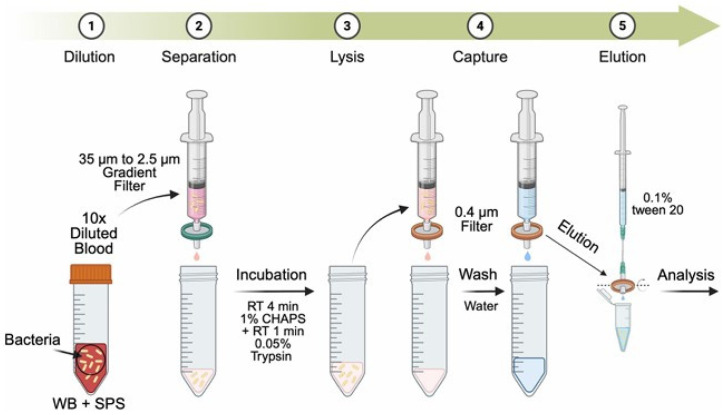
Dual-filter workflow for isolating bacteria from blood in 30 min and enabling downstream PCR assays with 5 steps [27]: (1) blood dilution and osmotic treatment; (2) gradient filtration to separate blood cells (35 μm to 2.5 μm); (3) chemical and enzymatic lysis of residual blood cells; (4) bacterial capture via 0.4 μm filter; (5) bacterial elution.

Despite size differences among red blood cells, white blood cells, platelets, and bacteria, directly filtering bacteria from blood remains challenging. Direct mechanical filtration effectively filters blood, yet its simplicity is challenged by red blood cell aggregation, forming a “filter cake” that obstructs blood and bacteria flow. This aggregation issue requires additional strategies to enhance filtration efficiency. Moreover, this approach cannot remove proteins and other biomolecules present in the blood. To address this issue, Park et al. proposed a dual-filter workflow enabling blood culture-free, sensitive detection of pathogenic bacteria from blood samples [27]. In this workflow, blood is first passed through a gradient filter (35–2.5 μm) to remove blood cells, debris, and aggregates. After chemical and enzymatic lysis of residual red blood cells, the filtrate is passed through a second 0.4 μm filter to capture bacteria, which are then recovered by reverse elution for downstream PCR and AST (Figure 1).

An alternative strategy is blood-lysis filtration, which employs detergents, salts, and/or proteases to lyse blood components (RBCs, WBCs, and platelets), so that only bacteria remain on the membrane surface. The selection of lysis solution is critical to achieve high efficiency in lysing blood elements while preserving bacterial viability, which presents a significant challenge. Wood et al. developed a novel whole blood filtration method for concentrating bacteria from bloodstream infections (BSIs) without the need for culturing blood or bacteria [28]. This method achieved complete bacterial recovery from 5 mL of whole blood in approximately 90 s. Building on prior studies investigating the effects of ionic/non-ionic detergents, Na_2_CO_3_, filter membrane surface, salt, and pH on lysis efficiency, they explored a new formulation using non-ionic detergents of Brij 58, Tween-80, and Pluronic F108 with NaCl solution. This was followed by filtration with a pre-soaked hydrophobic filter and backflushing with Pluronic F108, resulting in satisfactory separation and enrichment outcomes.

Due to the unique properties of blood samples, filtration-based methods still face significant challenges that require further innovative solutions. While using filtration alone can be difficult, combining filtration with other techniques, such as centrifugation, can greatly improve performance. In the context of microfluidic devices, integrating filtration and centrifugation into a single platform enables more efficient bacterial separation from blood samples. Such integrated approaches hold promise for enhancing the sensitivity and reliability of blood-based pathogen detection.

### 2.2. Bacteria Separation and Enrichment from Blood by Centrifugation

Centrifugation refers to a technique that separates components of a mixture by spinning them at high speeds, generating centrifugal force. Several factors, including particle size, shape, density, and the properties of the medium (viscosity, density, etc.), can influence the separation process during centrifugation. Usually, denser particles are forced outward, forming a compact “pellet” at the bottom of the tube, while lighter components remain near the axis of rotation, resulting in a supernatant layer above the pellet. As a result, centrifugation enables the rapid and efficient separation of complex mixtures and is widely used in various fields. As shown in Table 1, the densities of RBCs, WBCs, and platelets are within a similar range to that of bacteria, so standard centrifugation methods cannot effectively separate bacteria from blood components. However, because bacteria are much smaller than RBCs or WBCs, their sedimentation velocity is significantly lower. Platelets, with a size of ~3 µm, have a sedimentation velocity similar to or slightly higher than that of bacteria, which presents an additional challenge during the separation process. Therefore, the principles of sedimentation velocity can be leveraged to separate bacteria from blood cells by exploiting these substantial size differences. Over the past decade, researchers have investigated various centrifugation conditions (time, speed, medium, and multistep protocols), combined them with filtration and cell lysis, and explored integrated devices and hybrid techniques to enhance bacterial recovery while efficiently removing contaminants [29,30,31,32,33,34,35,36,37,38,39].

**Figure 2:**
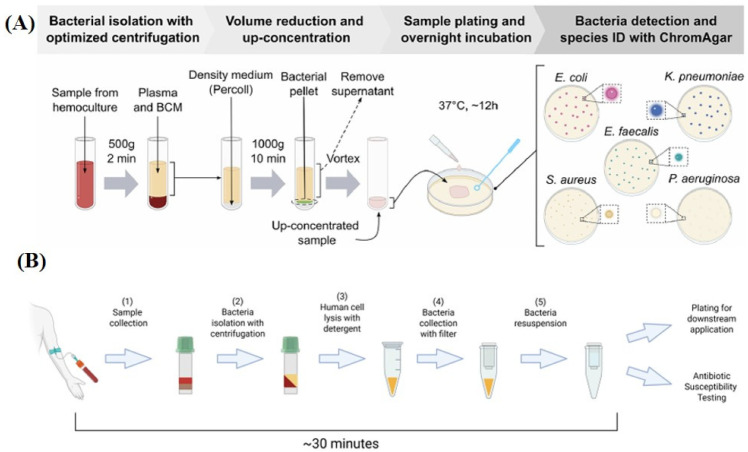
(**A**) A protocol for isolating, detecting, and identifying bacteria from blood involves optimized centrifugation, volume reduction with Percoll, sample plating and overnight incubation, followed by detection and species identification using chromogenic agar plates [29]. (**B**) A simple protocol enables pathogenic bacteria isolation from human blood in 30 min using centrifugation and standard laboratory equipment [30].

A method has been established for the isolation and identification of bacteria from blood within 12 h using a standard laboratory centrifuge and commonly available equipment, relying on optimized centrifugation parameters [29]. In the first low-speed, rapid centrifugation step, bacterial isolation takes advantage of the fact that bacteria have an approximately 30-fold lower sedimentation velocity than RBCs due to differences in size and hydrodynamic radius. The resulting supernatant, containing plasma and bacteria, is then supplemented with Percoll density medium and subjected to high-speed centrifugation for 10 min. The density medium acts as a “cushion” to minimize centrifugal compression of the bacterial pellet and facilitate the removal of remaining blood components (Figure 2A). Coskun et al. also reported a simple and fast protocol for isolation of pathogenic bacteria from human blood with over 70% bacteria isolation efficiency within 30 min [30]. It remained effective at low bacterial concentrations (1–10 bacteria per 0.3 mL of blood) and maintained bacterial viability without any significant change in growth lag times. In this work, a fast centrifuge step at 6000× *g* for 2 min in heparin tubes was used to remove blood cells and obtain plasma and bacteria, followed by lysis to further remove remaining blood cells. Bacteria filtration and centrifugation were performed to obtain pure bacteria for antibiotic susceptibility testing (Figure 2B).

**Figure 3:**
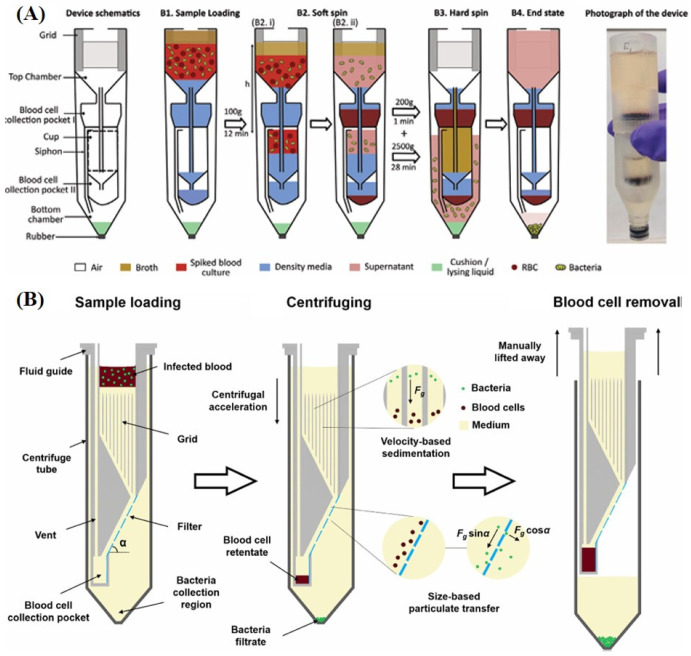
(**A**) Cross-sectional schematic, operation, and photograph of a centrifuge-based device for rapid, automated bacterial isolation from blood. A soft spin at 100 g settles blood cells without transferring liquid, followed by a hard spin that moves supernatant to the bottom chamber; stopping the centrifuge results in a small residual volume of bacteria in the bottom chamber [35]. (**B**) Cross-sectional schematic of a filter-in-centrifuge device consisting of a 3D-printed fluid guide with a filter inserted into a centrifuge tube for separating low-concentration bacteria from blood. Blood cells, which sediment faster and cannot pass the filter, collect in the pocket, while bacteria pass through the filter pores into the tube bottom [36].

The centrifugation method provides high throughput but is labor-intensive and prone to contamination due to its multiple downstream steps, heavily reliant on operator expertise. To address these limitations, Osaid et al. developed a plug-and-play centrifuge-only device for separating bacteria from blood, facilitating rapid sepsis diagnosis [35]. This 3D-printed device is designed in the form of a standard 50 mL centrifuge tube and consists of top and bottom chambers connected by a siphon. The bottom chamber is pre-filled with 0.4 mL of either lysing solution or cushion liquid, depending on the downstream application. The top chamber is first filled with density medium, followed by the spiked blood mixed with culture medium, and then broth medium is added on top. The device is then subjected to a soft centrifugation at 100 g, allowing blood cells to settle in the collection pocket while restricting liquid movement into the bottom chamber. Afterward, high-speed centrifugation is applied to move the supernatant into the bottom chamber and sediment the remaining blood cells and bacteria. Finally, the centrifuge is stopped, causing the liquid to transfer back from the bottom chamber to the top, leaving a small volume of liquid in the bottom chamber (Figure 3A). To overcome red blood cell-induced clogging in direct filtration, discussed in Section 2.1, Zeng et al. developed a method that separates bacteria from blood through a single centrifugation step, followed by size-based cross-flow filtration [36]. In this approach, a 3D-printed chip, referred to as a “fluid guide,” containing a filter was inserted into a centrifuge tube. The pore size of the filter was designed to be larger than bacteria but smaller than red blood cells. The filter within the fluid guide is inclined at an angle relative to the direction of centrifugal acceleration, facilitating efficient separation. Additionally, the region above the filter features a grid of vertical walls to suppress liquid convection caused by the Coriolis effect during centrifugation. Because the red blood cells are unable to pass through the filter, they are collected in a dedicated blood cell pocket, while the filtrate containing bacteria passes through to the lower region of the centrifuge tube (Figure 3B).

### 2.3. Bacteria Separation and Enrichment from Blood by Chemical Capture of Functional Magnetic Beads

The surface of bacteria is rich in various chemical components, such as proteins, polysaccharides, and lipids, which endow them with unique biological properties for recognition with a series of biomolecules [40]. The surface of Gram-positive bacteria mainly consists of a thick peptidoglycan layer and teichoic acids, and may be surrounded by a polysaccharide capsule. The surface of Gram-negative bacteria contains a thinner peptidoglycan layer and is covered by an outer membrane, which contains molecules such as lipopolysaccharides and outer membrane proteins. These components collectively determine the bacteria’s resistance, immune evasion, and interactions with the environment. Capturing bacteria by targeting specific chemical molecules on their surface enables efficient separation and enrichment. The substrates used for this purpose can take various forms, such as affinity columns, membranes, or magnetic beads. Among these, magnetic beads have been widely used in various areas, such as separation and enrichment, nucleic acid extraction, and more, due to their unique advantages of large surface area-to-volume ratio, high magnetic susceptibility, ease of surface modification, excellent biocompatibility, and compatibility with automated high-throughput operations [41,42]. Therefore, this section will focus on recent advances in the surface-modified magnetic bead-based chemical capture and separation of bacteria from blood. By modifying the surface of magnetic beads with different functional molecules, bacteria can be captured based on the specific molecules present on their surface for enrichment from a blood sample and subjected to downstream bacteria identification and AST [43,44,45,46,47,48,49,50,51,52,53,54,55,56,57,58,59,60,61,62]. Table 2 summarizes representative examples of functional molecule-modified magnetic beads and their corresponding bacterial surface targets for bacteria enrichment from blood. Selected cases using biomolecule-modified beads (e.g., MBL, FcMBL, IgG, SiO_2_, antibodies) for bacterial capture are discussed in detail in the following section. In addition to the functional molecules mentioned, biotechnology advancements promise to broaden the application of nanobodies, antimicrobial peptide sequences, and phage proteins, enhancing bacterial enrichment in blood diagnostics.

**Figure 4:**
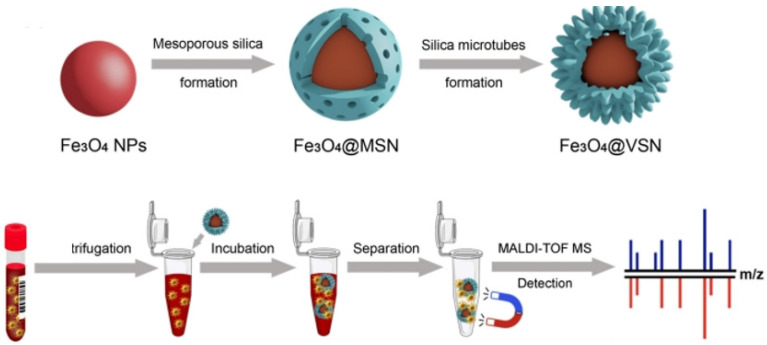
Schematic diagram depicting the preparation of Fe_3_O_4_@MSN and Fe_3_O_4_@VSN, as well as the enrichment of bacteria from blood by Fe_3_O_4_@VSN for MALDI-TOF MS identification [44].

Based on the nonspecific interactions between bacterial cells and the surface of SiO_2_-encapsulated Fe_3_O_4_ nanoparticles, Zhang et al. demonstrated that magnetic nanoparticles with a viral spiked structure (Fe_3_O_4_@VSN) exhibited excellent binding affinity toward both Staphylococcus aureus and Klebsiella pneumoniae isolated from clinically positive blood cultures (Figure 4) [44]. Since mannose-binding lectin (MBL) can recognize and bind to mannose, fucose, and N-acetyl-D-glucosamine residues present on the surfaces of various bacteria, MBL has also been conjugated to magnetic beads to facilitate the enrichment of common bacterial pathogens from blood [45]. MBL exhibits broad-spectrum binding to both Gram-negative and Gram-positive bacteria but shows low affinity for certain species, such as *E. coli*. To enhance bacterial binding, FcMBL was engineered by fusing the MBL neck and carbohydrate recognition domains with the human IgG Fc region, improving expression, purification, and reducing coagulation/complement activation. FcMBL-conjugated magnetic beads also show broad-spectrum bacterial capture from blood, and with higher affinity for some bacteria than MBL-conjugated beads [46,47,48,49,50]. Human IgG binds to protein A, protein G, protein L, and glycans on bacterial surfaces, also enabling the recognition of various bacterial species. Yi et al. demonstrated this method’s feasibility by enriching and identifying six pathogenic bacteria, and using IgG@Fe_3_O_4_ enrichment and MALDI-TOF MS, bacteria at concentrations as low as 10^5^ CFU in 100 μL of human whole blood were successfully detected [51]. To leverage the distinct binding mechanisms of Fc-MBL and IgG, Xue et al. developed a strategy utilizing co-magnetic beads coupled with Fc-MBL@Fe_3_O_4_ and IgG@Fe_3_O_4_ [52]_._ This combined approach demonstrated higher bacterial capture efficiency than using either FcMBL@Fe_3_O_4_ or IgG@Fe_3_O_4_ beads individually. Kim et al. utilized magnetic nanoparticles decorated with synthetic beta-2-glycoprotein I (sβ2GPI) peptides to enable blood culture-free, ultra-rapid antimicrobial susceptibility testing. β2GPI is an acute-phase plasma protein known to play a role in human innate immune responses by naturally recognizing common motifs of pathogen-associated molecular patterns, thereby allowing the capture of a broad range of blood-borne pathogens [53]. The synthesized 1-(12-(mercaptododecyl)-3-methylimidazolium bromide (MDMIBr) ionic liquid was also immobilized on Fe_3_O_4_ nanoparticles for broad-spectrum bacteria capture capability for blood purification [54]. In addition, in recent years, phenylboronic acid and its derivatives have garnered significant attention as recognition molecules for bacterial detection, owing to their selective binding to cis-diol groups present on bacterial surfaces. [55,56] However, there have been very few reports on their application for isolating bacteria from blood samples, indicating substantial potential for further development and utilization in this area.

**Figure 5:**
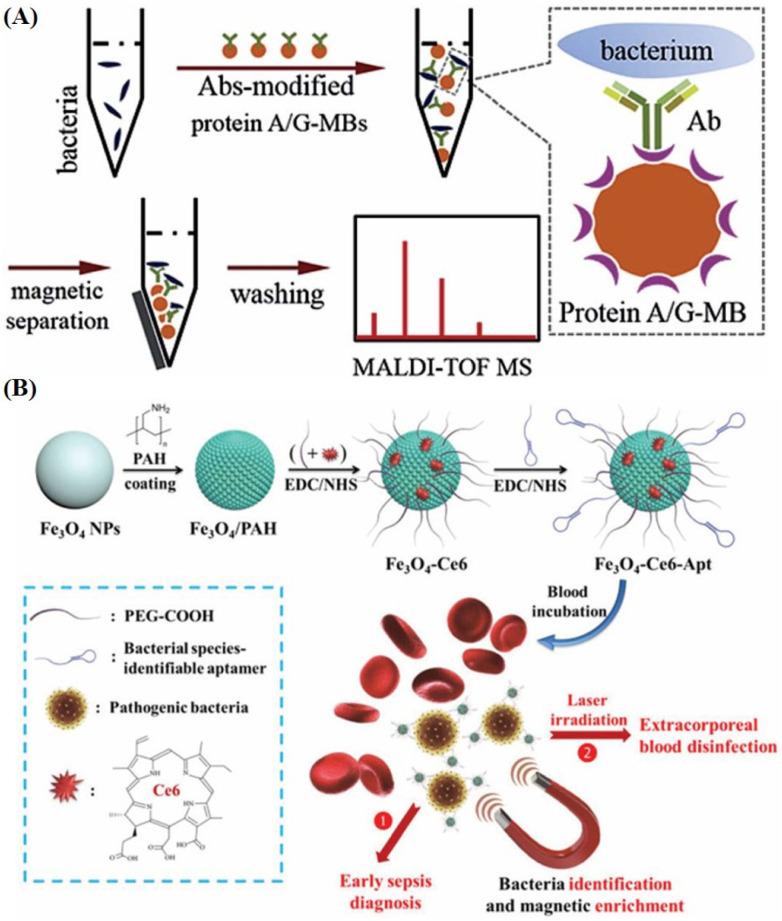
(**A**) Schematic representation of the immunoaffinity MALDI-TOF MS procedure utilizing antibody-modified magnetic beads to capture bacteria from blood [57]. (**B**) Preparation and conceptual illustration of the Fe_3_O_4_-Ce6-Apt nanosystem for magnetic enrichment of bacteria and their identification for early sepsis diagnosis [60].

The above paragraph discussed the use of functional molecules conjugated to magnetic beads to enable broad-spectrum bacterial capture, facilitating the simultaneous isolation and enrichment of a wide range of bacterial types. This is the most common application, as the identity of invading pathogens in blood is usually unknown; thus, enrichment methods must be able to cover a wide range of bacterial strains while keeping the microorganisms viable and intact for subsequent analysis. On the other hand, in situations where the bacterial species is already known or there is an urgent need to detect a particular pathogen, high-affinity and high-specificity capture strategies utilizing antibodies or aptamers can be considered. And in certain circumstances, it may be advantageous to employ both broad-spectrum and targeted bacterial capture approaches. The antibody-modified magnetic bead demonstrated highly efficient enrichment and separation of certain bacteria from blood samples due to the strong affinity between antibodies and their corresponding antigens on bacteria surface (Figure 5A) [57]. In addition, numerous aptamer sequences that recognize a wide range of bloodstream infection (BSI) pathogens have also been reported through in vitro selection using the systematic evolution of ligands by the exponential enrichment (SELEX) process, and demonstrated their application for bacteria enrichment via magnetic separation [59,60,61,62]. In most cases, these aptamers exhibit high selectivity for a single bacterial species; however, there are also aptamers with an expanded host range of bacteria. For example, Wang et al. developed an Fe_3_O_4_-Ce6-Apt nanosystem, consisting of iron oxide nanoparticles functionalized with chlorin e6 and bacteria-specific aptamers, for early sepsis diagnosis and extracorporeal blood disinfection, enabling one-step bacterial identification/enrichment and fluorescence detection of mono-(*S. aureus*) or polymicrobial (*S. aureus*, *E. coli*) infections in mice (Figure 5B) [60].

It should be noted that, in contemporary studies, magnetic beads used for capturing and enriching bacteria from blood samples are typically applied directly in subsequent bacterial identification and antimicrobial susceptibility testing. The process of releasing bacteria from the magnetic beads prior to further detection has not yet received significant attention. Sun et al. demonstrated that releasing bacteria from functional magnetic beads can affect MALDI-TOF MS identification results [50]. They constructed FcMBL@Fe_3_O_4_ beads for bacterial enrichment, as FcMBL binds bacteria in a Ca^2+^-dependent manner. Since Ca^2+^-EDTA has a much higher affinity than Ca^2+^-FcMBL (Kd ~10^7^ M vs. Kd ~10^−3^ M), adding EDTA effectively releases bacteria from the beads. Their findings showed that functional materials, especially proteins, may interfere with MS signals and cause identification errors. Therefore, releasing bacteria from these materials significantly improves the accuracy of MALDI-TOF MS database-based identification. Therefore, releasing bacteria from magnetic beads can impact subsequent mass spectrometry analysis, molecular amplification, and various antimicrobial susceptibility testing methods. The release method and efficiency are closely related to the specific binding mechanisms used, making this an important issue that warrants further attention and research.

### 2.4. Bacteria Separation and Enrichment from Blood by Microfluidic Chips

Benefiting from advances in micro/nanofabrication, materials, and detection technologies, microfluidic chip technology has evolved from simple “microchannel” into fully integrated lab-on-a-chip systems that combine sampling, mixing, separation, detection, and even data processing within a single platform [63]. For separating and enriching bacteria from blood, microfluidics offers several distinct advantages: (1) It enables precise control of flow fields in microliter-scale volumes and can achieve physical separation of bacteria from blood cells through various mechanisms. (2) It readily integrates with optical, electrochemical, and molecular diagnostic modules to realize “separation + detection” on a single chip, thereby significantly shortening the time required for BSI diagnosis. (3) It supports the development of disposable, low-cost devices, which are particularly advantageous for point-of-care testing and for use in resource-limited settings. Over the past decade, numerous studies have employed microfluidic chips that exploit acoustic fields, dielectrophoresis, magnetic fields, size differences, or inertial flow to separate and enrich bacteria from blood [64,65,66,67,68,69,70,71,72,73,74,75,76,77,78,79,80,81]. Bacterial detection modules have also been integrated into these chips, further demonstrating the advantages of microfluidic chip-based approaches for bacterial enrichment from blood and the rapid diagnosis of BSIs.

**Figure 6:**
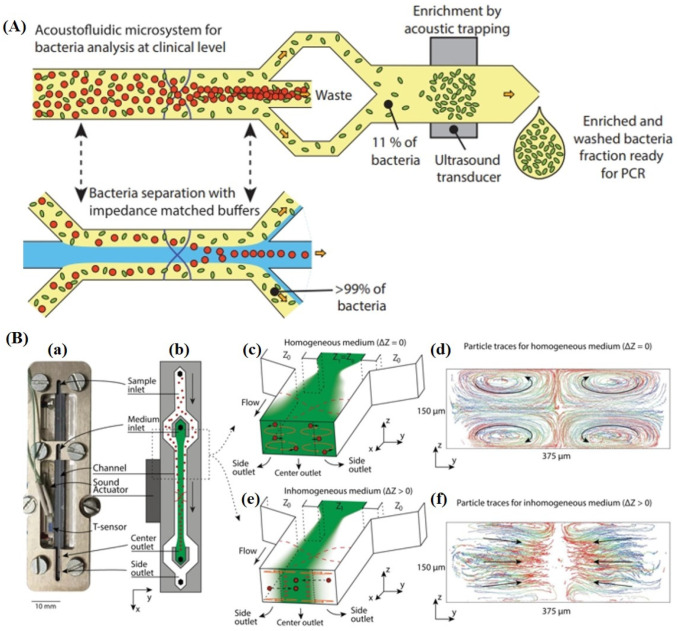
(**A**) With conventional buffers, acoustic deflection of red blood cells can entrain nearby bacteria via hydrodynamic drag, causing co-migration and low bacterial recovery (~11%). Using the impedance-matched buffer described here minimizes this coupling and boosts bacterial recovery to >99% [64]. (**B**) Working principle of gradient acoustic focusing (GAF) [65]: (**a**) Photograph of the microfluidic chip mounted in its holder. (**b**) Schematic of the GAF separation concept. (**c**) When the acoustic impedances of the central medium (Z_1_) and the particle suspension (Z_0_) are matched (ΔZ = 0), acoustic streaming induces mixing. (**d**) 3D-tracked positions of 1 µm particles during 20 s of stopped flow for ΔZ = 0, showing streaming-driven mixing; color indicates time from 0 s (blue) to 20 s (red). (**e**) Injecting a higher-impedance Ficoll solution through the central inlet creates an impedance gradient (ΔZ > 0) that suppresses streaming and enables particle translocation into the central stream. (**f**) Corresponding 1 µm particle tracks for ΔZ > 0, showing suppressed streaming; color indicates time from 0 s (blue) to 20 s (red).

The acoustic microfluidic (acoustophoresis) applies a standing ultrasonic wave across a microchannel to generate acoustic radiation forces that drive suspended particles to specific pressure nodes or antinodes. Because the acoustic radiation force and the resulting migration velocity depend strongly on particle size and acoustic contrast, the size disparity between bacteria and blood cells produces distinct lateral migration behaviors, enabling continuous, label-free, size-based separation of bacteria from blood [64,65,66,67,68,69]. This approach simplifies sample preparation and helps preserve cell viability and proliferative capacity, which is beneficial for downstream culture of the separated bacteria. Moreover, acoustic platforms can be readily integrated with diverse detection modalities to enable integrated microfluidic analysis systems. Ohlsson et al. showed that using acoustic impedance-matched buffers can maintain effective separation of bacteria from blood cells even at high blood cell concentrations [64]. In conventional buffers, the higher acoustic impedance of blood (due to cells and plasma proteins) can cause the entire sample stream to shift, and hydrodynamic coupling with blood cells can drag nearby bacteria, reducing separation efficiency. By matching the acoustic impedance of the central medium, they processed 1 mL of undiluted whole blood in 12.5 min, achieving ~90% bacterial recovery while removing >99% of blood cells (Figure 6A). To extend acoustic focusing below the particle size limit of ~2 µm, Van Assche et al. developed gradient acoustic focusing (GAF), which uses a co-flowing acoustic impedance gradient to generate a stabilizing acoustic volume force that suppresses acoustic streaming and drives particles across media [63]. They demonstrated continuous focusing of multiple bacterial species and efficient medium exchange, enabling chip-integrated sample preparation by separating bacteria from blood lysate for downstream detection (Figure 6B).

**Figure 7:**
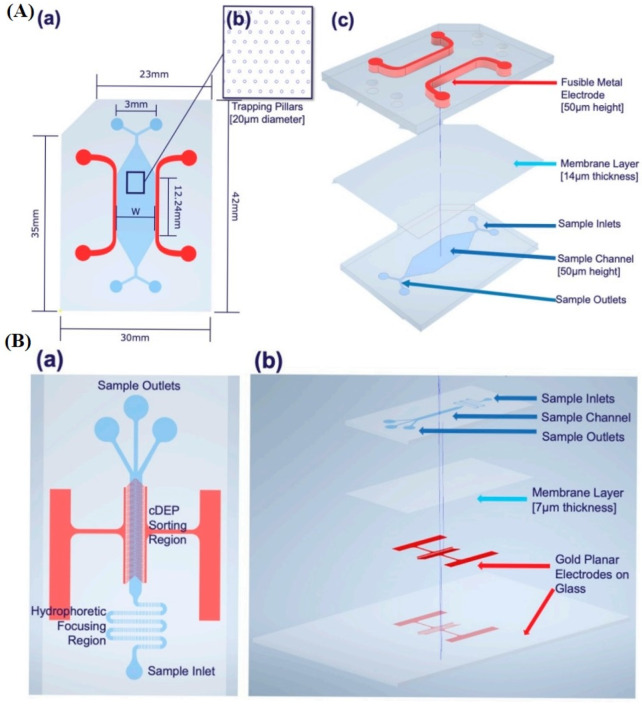
Geometry and design features of CytoChip B and CytoChip D [75]. (**A**) CytoChip B. (**a**) The width between the electrodes, w, was held constant at 3.4 mm. (**b**) Close-up of the pillars located in the sample channel, and (**c**) the three-layer stack-up. (**B**) CytoChip D. (**a**) Top view of the assembled multi-layer device. (**b**) Illustration of the multi-layer channel, membrane, and electrode stack-up.

Dielectrophoretic (DEP) microfluidic separation often uses electrodes to generate a non-uniform electric field in a microchannel to polarize particles and generate a net force even if they carry no net charge. By tuning the AC frequency to adjust particle polarizability relative to the medium, different particles experience either positive DEP (toward high-field regions) or negative DEP (toward low-field regions), producing distinct lateral displacements in laminar flow and enabling outlet-based separation [70]. Several dielectrophoresis (DEP) device modalities have been developed for bacterial separation from blood, including electrode-based DEP (eDEP), insulator-based DEP (iDEP), optically induced DEP (oDEP), and contactless DEP (cDEP), etc. [71,72,73,74,75]. In cDEP, an AC voltage is applied to electrodes that are isolated from the sample channel by an insulating barrier (e.g., PDMS or glass), so the electric field is coupled into the fluid without direct electrode contact; this mitigates electrolysis/bubble formation and electrode fouling, helping preserve bacterial viability and native phenotypes. Using cDEP, Thomas et al. compared two label-free sorters (CytoChip B and CytoChip D) for throughput, scaling, precision targeting, and high-viability recovery for enriching *E. coli* from whole human blood (Figure 7) [75]. The results demonstrated that CytoChip D offered higher potential throughput for large samples (e.g., 10 mL) but showed poor bacterial recovery due to insufficient hydrophoretic focusing, whereas CytoChip B achieved high-purity enrichment with >98% viable recovery even at <100 CFU/mL, though scaling will likely require parallelization/longer channels and integration with upstream bulk methods (e.g., deterministic lateral displacement or RBC lysis). Overall, optimizing chip architecture and integrating complementary label-free techniques are key directions for translating DEP-based platforms toward rapid bacterial enrichment and BSI diagnostics.

**Figure 8:**
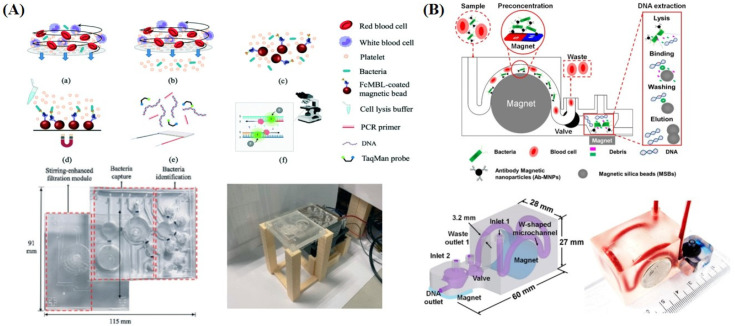
(**A**) Workflow and images of an integrated microfluidic chip for bacterial isolation and identification from human blood [78]. The process includes the following steps: load bacteria-spiked blood; separate bacteria-enriched plasma from non-target cells; transfer isolated bacteria to a micromixer and gently mix with FcMBL-coated magnetic beads; magnetically collect bead-bacteria complexes; PCR amplify bacterial DNA; and detect fluorescence with a PMT for identification (**a**–**f**). (**B**) Schematic, design overview, and photo of a 3D-printed modular microfluidic device (3DpmµFD) for bacterial preconcentration and genomic DNA extraction from blood [79]. *E. coli* O157:H7 is magnetically enriched in a microchannel using serotype-specific antibody-magnetic nanoparticle conjugates and a permanent magnet, then transferred to a conical microchamber for lysis and gDNA capture on silica MSBs in chaotropic salts (DNA extraction).

The previous section described the use of functional magnetic beads to isolate and enrich bacteria from blood. This strategy can be readily integrated into microfluidic chips to enable automated operation, and several studies have further demonstrated the combination of on-chip bacterial detection and the separation process, highlighting the “sample-in, answer-out” advantage of integrated microfluidic platforms [76,77,78,79]. Fang et al. developed an integrated microfluidic chip that isolates and identifies both Gram-positive and Gram-negative bacteria from blood [78]. The device integrates membrane filtration, micromixer-based capture with protein A-FcMBL-coated magnetic beads, and on-chip TaqMan PCR, achieving 100% WBC removal, 99.5% RBC depletion, 56–85% capture of five bacterial species within 20 min, and detection down to 5 CFU/mL in ~4 h (Figure 8A). The capture efficiency could be further improved by optimizing filtration and bead-binding conditions. Abafogi et al. reported a 3D-printed modular microfluidic device (3DpmµFD) that concentrates bacteria from whole blood and purifies their genomic DNA (gDNA) using a W-shaped capture channel and a conical extraction chamber [79]. The bacteria are captured in the channel with antibody-conjugated magnetic nanoparticles (Ab-MNPs), after which a 3D-printed rotary valve connects the conical chamber to extract gDNA using magnetic silica beads (Figure 8B). The workflow processes 2.5 mL of spiked blood in ~50 min and, combined with PCR/qPCR, detects *E. coli* O157:H7 down to 10 CFU/mL.

Beyond the approaches discussed above, microfluidic platforms have also been used to isolate and enrich bacteria from blood by leveraging physical mechanisms such as elasto-inertial microfluidics [80,81]. In summary, microfluidic chips offer a compelling platform for separating and enriching bacteria from whole blood because they integrate sample handling, separation, and detection preparation into a compact, low-dead-volume device, enabling faster time-to-result than culture and reducing reliance on bulky laboratory infrastructure. Their precise control of microscale flows allows exploitation of subtle biophysical differences such as size, deformability, density, surface affinity, and electrical properties, so bacteria can be enriched while many blood components are depleted, improving downstream sensitivity for PCR, sequencing, or rapid phenotypic assays. However, several challenges remain to be addressed, including ultra-low bacterial counts requiring high recovery at clinical throughput; complex blood matrices that cause clogging, nonspecific adsorption, and hemolysis; and poor discrimination from similarly sized debris/vesicles. Performance must be robust across patient variability, anticoagulants, and storage.

Future microfluidic-based bacteria enrichment platforms from blood will likely evolve into integrated, multi-stage sample-to-answer systems that combine hybrid separation, antifouling high-throughput design, and on-chip lysis/inhibitor removal with standardized performance and compatibility with downstream phenotypic or molecular analysis. It is worth noting that droplet microfluidics, which enables the high-throughput generation and manipulation of subnanoliter droplets, has become a powerful tool in modern chemical and biological research [82,83,84,85]. In integrated sample-to-answer microfluidic platforms for bacterial enrichment and BSI diagnosis, droplet microfluidics may serve as an enabling layer linking upstream enrichment with downstream analytical steps. Although not yet widely applied in this specific area of bacterial enrichment, droplet microfluidics has shown clear advantages when combined with other microfluidic separation methods, such as acoustofluidics and dielectrophoresis [86,87,88]. It is envisioned that active droplet handling after microfluidic enrichment can facilitate sub-sampling, reagent exchange, modular reaction sequencing, and other microscale processing tasks requiring precise volumetric control and low reagent consumption, thereby playing an important role in future diagnostic systems.

### 2.5. Comparative Analysis of Four Approaches for Bacterial Separation and Enrichment from Blood in BSI Diagnostics

The above section introduces and discusses four major approaches for bacterial separation and enrichment from blood in the diagnosis of BSIs, including filtration, centrifugation, functionalized magnetic beads, and microfluidic chips. Filtration relies on size exclusion and is simple and low-cost, but is hindered by red blood cell deformability and membrane clogging. Centrifugation separates bacteria by sedimentation differences, yet overlapping properties with blood cells often require multistep optimization. Functionalized magnetic beads capture bacteria via molecular recognition and are compatible with MALDI-TOF MS, although bacterial release can be challenging. Microfluidic chips exploit acoustic, dielectrophoretic, inertial, and magnetic forces, enabling integrated and automated sample-to-answer workflows. Given the substantial heterogeneity in experimental conditions, direct quantitative comparison among different separation strategies is challenging. Therefore, Table 3 provides a qualitative summary of the four major bacterial separation and enrichment methods and offers a comparative framework.

As indicated in Table 3, each method is characterized by multiple performance metrics, such as recovery, purity, throughput, etc. Accordingly, these approaches exhibit distinct advantages and limitations. Table 4 further compares the four methods in terms of their underlying principles, strengths, and weaknesses.

## 3. Translational Considerations and Clinical Implementation Challenges of Bacterial Separation and Enrichment Methods

Recent advances in bacterial separation and enrichment have improved the feasibility of bloodstream infection diagnosis by providing a key front-end step for pathogen identification and AST. Yet, clinical translation depends not only on recovery efficiency, but also on cost, throughput, robustness to complex blood, and workflow integration. Different strategies also vary in compatibility with downstream applications. Filtration, centrifugation, microfluidic-based acoustic separation, or dielectrophoresis are viability-preserving methods with high bacteria recovery and are more suitable for culture-based analysis and phenotypic AST. On the other hand, affinity-based functional magnetic beads are often better aligned with molecular assays or MALDI-TOF MS identification. Microfluidic chips can be rationally designed with tailored structures and functionalities to meet different diagnostic objectives. For bacterial species identification, microfluidic platforms can integrate separation with downstream lysis, nucleic acid extraction, and amplification, enabling a sample-to-answer workflow. In terms of cost and scalability, filtration and centrifugation are relatively simple and can be performed with standard laboratory equipment, but they often require multiple manual steps, such as cell removal, washing, density-based separation, and elution, which makes automation more difficult. Functionalized magnetic beads are more amenable to automated and high-throughput workflows because of their magnetic properties and easy surface modification, although their practical use may require additional preparation effort. Microfluidic chips offer the greatest integration potential, as they can combine separation and detection on a single platform; however, their broader clinical translation will depend on scalable manufacturing, device standardization, and further reduction in per-unit cost.

Reported recoveries vary substantially across platforms rather than uniformly exceeding 80%. In general, physical methods such as filtration and centrifugation are attractive for complex samples because they are label-free, broadly applicable, and compatible with standard laboratory equipment. These approaches may be particularly useful when the clinical sample is polymicrobial or contains very low bacterial burdens, since preserving viable organisms for culture-based analysis, follow-up identification, or AST can be advantageous. However, their performance is often constrained by blood cell interference, multistep processing, and loss of rare bacteria during handling. Functionalized magnetic beads and microfluidic chips offer stronger integration and automation, but their practical use still depends on efficient recovery from whole blood, low nonspecific loss, and compatibility with downstream assays such as PCR, MALDI-TOF MS, sequencing, and phenotypic AST. Overall, the main barriers to routine clinical implementation include not only insufficient recovery and purity but also poor standardization, workflow complexity, clogging, matrix interference, and inconsistent compatibility with downstream identification and susceptibility testing; these issues remain important challenges to be addressed in future work.

## 4. Outlook and Future Perspective for Bacteria Separation and Enrichment from Blood

Significant progress has been made in bacterial separation and enrichment from blood, yet several key gaps continue to limit routine clinical translation. The most important challenges remain the extremely low bacterial burden in early bloodstream infection, interference from complex blood matrices, and the lack of robust, standardized, cost-effective, sample-to-answer workflows. From the current research trend, future development of bacterial separation and enrichment technologies should focus on several key directions. First, efficient handling of larger-volume blood samples within shorter timeframes should be achieved through parallelized designs, clog-resistant structural optimization, and integration of pretreatment modules. Second, multi-stage hybrid separation platforms should be developed by combining different mechanisms, such as inertial, acoustic, dielectrophoretic, magnetic, and affinity-based capture, so as to balance broad-spectrum capture capability, selectivity, and biocompatibility, thereby meeting the separation requirements of Gram-positive bacteria, Gram-negative bacteria, and bacteria in different physiological states. Third, material and surface engineering should be further strengthened, including antifouling coatings, low-adsorption materials, and disposable device designs, to reduce nonspecific loss, blood contamination, and device biofouling, while preserving bacterial viability and biomolecular integrity as much as possible to satisfy the requirements of rapid pathogen identification and AST. In addition, standardized evaluation criteria are essential for the objective comparison of bacterial separation and enrichment platforms. Although existing methods differ in recovery, purity, processing time, sample compatibility, and downstream assay performance, unified metrics remain lacking. Future work should therefore emphasize standardized experimental design, performance assessment, and clinical validation to define the strengths and limitations of each approach. Overall, bacterial enrichment from blood should be viewed as a core front-end component of rapid BSI diagnostics, enabling integrated pathogen identification and AST. With continued advances in micro/nanofabrication, materials science, and bioanalysis, clinically deployable strategies characterized by a high throughput, low bias, low contamination, and robust standardization are expected to accelerate the translation of BSI diagnostics.

## Figures and Tables

**Table 1:** Representative physical properties of components and bacteria in blood.

Component	Density Range (g/cm^3^)	Estimated Size (μm)	Concentration (Cells/mL)
Red blood cells (RBCs)	1.086–1.122	7.5–10.0	4.0–5.7 × 10^9^
White blood cells (WBCs)	1.057–1.092	7.0–20.0	3.0–11.7 × 10^6^
Platelet	1.072–1.077	1.5–3.0	2.0–4.0 ×10^8^
Plasma	1.024	/	Containing high-concentration proteins and other molecules
Bacteria	1.08–1.10	0.8–3.0	1–100 in early stage of infection

**Table 2:** Representative examples of functional molecule-modified magnetic beads for the separation and enrichment of bacteria from blood.

Capture Biomolecules	Targeted Molecules on Bacteria Surface	Target Bacteria	Blood Volume (mL)	Capture Time (min)	LOD (CFU/mL)	Ref
SiO_2_	nonspecific adsorption	*S. aureus*, *K. pneumoniae*	0.2	60	10^5^, 10^4/^MALID-TOF MS	[44]
mannan-binding lectin (MBL)	mannose, fucose, N-acetyl-D-glucosamine residues	*S. aureus*, *P. aeruginosa*, *A. baumannii*	10	>60 min	10/PCR	[45]
FcMBL	mannose, fucose, GlcNAc, PAMPs	*K. pneumoniae*, *P. aeruginosa*, *P. putida*, *E. faecal*, *etc.*	1	5	10^3^/MALID-TOF MS	[46]
human IgG	protein A, protein G, protein L, glycans	*S. aureus*, *K. pneumoniae*, *P. aeruginosa*	0.1	30	10^6^/MALID-TOF MS	[51]
co-magnetic bead with FcMBL and IgG	polysaccharides, Protein A/G/L	*S. aureus*, *S. capitis*, *E. coli*, *K. pneumoniae*	1	20	10^3^/MALID-TOF MS	[52]
sβ2GPI peptide	pathogen-associated molecular patterns (PAMPs)	18 clinical pathogens (G+/G−/fungi)	5–10	≤60	≤4/rapid AST	[53]
antibodies	peptidoglycan/Gram-positive surface	*S. aureus*, *E. faecium*, *B. cereus*	1	30	5 × 10^2^/MALID-TOF MS	[57]

**Table 3:** Systematic cross-method evaluation of four bacterial separation and enrichment approaches.

	Filtration	Centrifugation	Functionalized Magnetic Beads	Microfluidic Chips
Bacteria recovery (sensitivity)	High (can be >90%); loss by nonspecific adsorption	High (can be >90%)	Moderate to high (can be >90%)	Moderate to high (can be >90%); dependent on chip design and operating mode.
Purity of the enriched fraction	Moderate to high; proteins and biomolecules remain	Moderate to high; often needs lysis or filtration	Moderate to high; limited by nonspecific adsorption, and bead materials may interfere without release	High.
Preservation of bacterial viability	Excellent	Excellent	Good	Excellent.
Species breadth	Highly broad, no selectivity	Highly broad, no selectivity	Moderately broad, can be specific	Can be highly broad or specific.
Tolerance to low bacterial burden	High	High	Moderate	High.
Susceptibility to clogging or fouling	Moderate to high; RBC “filter cake” may cause blockage	High	High	Moderate to high; limited by channel block.
Throughput	Moderate; challenge for automation	High; multiple samples can be processed in parallel with standard centrifuges.	Low to high; limited by multiple steps, potential for automation	Low to high; parallelization needed for scale-up.
Blood volume	Broad; 0.1–10 mL	Broad; 0.1–10 mL	Broad 0.1–10 mL	10 μL–10 mL; high volume limited by flow rate.
Processing time	10–60 min	10–60 min	10–60 min	10–60 min, depending on flow rate.
Compatibility with downstream phenotypic AST or molecular detection	High with both; suitable for culture-based method	High with both; suitable for culture-based method	Moderate with AST, high with identification	Moderate to high; depends on function design
Sample preparation complexity	Moderate to high	Moderate to high; multistep and user-dependent	Moderate; requires functionalization, separation, and sometimes elution	High in device design, low in operation after integration.
Overall practicality for clinical implementation.	Moderate; low-cost but clogging and incomplete cleanup limit robustness	Moderate to high; accessible equipment, but workflow remains laborious	High potential; automation-friendly but standardization needed	Highest long-term potential; scalability and robustness remain challenges.

**Table 4:** Comparison of four bacterial separation and enrichment approaches in terms of principles, advantages, and disadvantages.

	Filtration	Centrifugation	Functionalized Magnetic Beads	Microfluidic Chips
Principle	Size exclusion filtration through membrane pores, enhanced by washing, lysis, and gradient or dual-filter processing.	Exploits sedimentation velocity differences, often combined with density media, lysis, filtration, or multistep protocols.	Surface-modified magnetic beads capture bacteria via specific recognition of bacterial surface targets.	Use microscale flow control and physical forces to separate and enrich bacteria.
Advantages	Label-free, simple, relatively low cost, scalable, and easy to combine with standard downstream assays.	Uses routine laboratory equipment, is easy to implement, and is suitable for mL-scale processing.	Provides high selectivity or broad-spectrum capture, good automation potential.	Enables high integration, precise fluid handling, low reagent consumption, and linkage of separation with detection and AST.
Disadvantages	Susceptible to membrane clogging, fouling, and nonspecific bacterial loss; requires optimization of lysis and pore size.	Limited selectivity; blood debris may co-sediment with bacteria; often involves multiple manual steps.	Performance depends on ligand chemistry; may introduce pathogen bias, increase cost, and complicate release.	Device fabrication and operation can be complex; standardization and clinical translation remain challenging.
Performance metrics	Provides rapid bacterial enrichment from blood for AST and PCR, yet RBCs’ deformability and filter clogging hinder recovery; gradient and lysis-assisted filtration improve low-abundance pathogen capture.	Separates bacteria from blood by exploiting sedimentation differences, but overlapping densities of bacteria and blood components limit efficiency; optimized multistep protocols and density media can enhance recovery.	Enables broad-spectrum or targeted bacterial capture from blood, but release efficiency and signal interference remain concerns.	Enables integrated, rapid bacterial enrichment from blood with reduced blood cell interference, but clogging, nonspecific adsorption, and poor performance in ultra-low bacterial burdens remain key challenges.

## Data Availability

No new data were created or analyzed in this review.
